# Sociology and Criminology

**Published:** 1896-01

**Authors:** Clark Bell

**Affiliations:** New York, President Medico-Legal Congress


### Society Notes.

### Sociology and Criminology.

BY CLARK BELL, ESQ., OF NEW YORK,
President Medico-Legal Congress.

[Address at Opening, Department of Sociology and Criminology, at the Medico-Legal Congress, New York, Sept. 5, 1895.]

That public interest in sociological studies, is on the increase in our day, can not be questioned. We may recognize its hold on popular thought, by remarking that in the present congress more papers are contributed to the department of sociology and criminology, than to any other. This is not in my opinion the result of chance, but is due to the increasing interest manifested in the world of thought in this domain of scientific investigation.

Especial stimulus has been given to the study of criminology by Lombroso's writings, and others of his school.

Enricco Ferri has touched it with his brilliant lance. Herman Kornfeld, Morris Benedikt, Kraft Ebbing, Morel, Le Grand du Saulle, Brierre du Boismont and Prosper Depine, on the continent; Havelock Ellis, W. W. Ireland, Pritchard, Thomas, Dr. Nicholson and W. Douglass Morrison in Great Britian, and many other writers of distinction have illumed its importance. The International Congress of Penal Law, attracted twice, many great names from all the world, to this subject.

It is not crime alone that we now study, with methods of punishment, sentences, prisons and their management, including discipline and corporal punisment in prisons, all the offenses, and the modifications in penal statutes; but we are coming to study more the criminal himself, his characteristics, degeneracy, heredity, and above all, environment.

Are punishments for crime, as defined in our penal statutes, really deterrent?

Has the State the moral right to inflict punishment in any retaliatory spirit as is now often times the basis of penal statutes? If experience demonstrates that excessive or prescribed forms of punishment do not act in fact as a deterrent in diminishing crime, should we not consider with greater care what modifications are proper to reach the end desired, besides the protection of society, a perceptible decrease in the volume of crime?

The lesson of the repeal of the long list of capital crimes in Great Britain since the day when sheep stealing was a capital offense, must not be lost. Severity of punishment does not appear to operate as a deterrent.

It seems to be true that the fear of the scaffold rarely deters the murderer.

Crime seems in the ocean of humanity, to be the sum of social causes, which like great rivers flow towards and empty into it. Its *Amazon* is no doubt alcoholic stimulants, which, more than all other causes combined, constitutes the inevitable, terrible, irresistible scourge of the race.

In its currents, tides and eddies, are insanity, epilepsy and physical degeneracy, not always in the parent, but more certainly, in the offspring.

Its movements run like the blood of man into the veins and lives of children's children, with a taint as terrible as that of leprosy or syphilis.

The burdens to the State for the care of the insane, in the rural districts or counties, notably in an agricultural county like *Yates*, where I reside in the summer, in this year of grace, is actually greater than the cost of the schools, and almost equal to the entire other expense of the State government, including the canals.

When will we have the courage to look this awful question squarely in the face, and decrease the volume of crime, not by penal laws for the puuishment of the criminal (often the victim of his birth and environment) but by striking at and repressing the cause?

The recognized defects in our penal laws, especially in Great Britain, the United States and many of the Continental States, may be summarized as follows:

1. The principle of equality of sentences, as to their duration as now existing, is erroneous and vicious in its fundamental principles.

2. It is wrong to make arbitrary punishments for the same offences against all offenders alike.

3. Criminal laws must be so framed as to meet the social conditions of the criminal classes.

Laws based upon the social conditions of men in the ordinary walks of life, fail. They should rather be aimed at the social life and condition of the criminal classes. I quite agree with W. Douglass Morrison, of the Wadsworth prison in England, when he asserts that "The criminal is a product of anomalous biological conditions, as well as adverse circumstances."

4. Some plan should be devised in the administration of punishments to offenders, under which the principle of determinate sentences should be applicable to the individual condition of the offender, for example:

a. The same offense should not receive the same punishment in all cases, as for example, when committed by an adult, or a child, or a youthful offender.

b. The difference in punishment for the same offense by a man and a woman, should be rather to *the* man and *the* woman.

5. We must consider whether Bentham was right in insisting that we should in adjusting our methods of punishment, look as much to the nature and condition of the offender, as to the nature of the offense.

Much of the failure of our present system as a protection to society, is unquestionably due to our ignoring this fundamental law, in our present penal statutes, and punishment of criminals Mr. Morrison strikes at an important principle when he claims that we should place our prisons on the same basis as the penal laws. That prisons should reach the causes and conditions which produce the criminal, and the penal statutes be placed on the same plane.—(From advance sheets *Medico-Legal Journal* and the *Bulletin of the Medico-Legal Congress.*)

